# Differentiation of IncL and IncM Plasmids Associated with the Spread of Clinically Relevant Antimicrobial Resistance

**DOI:** 10.1371/journal.pone.0123063

**Published:** 2015-05-01

**Authors:** Alessandra Carattoli, Salome N. Seiffert, Sybille Schwendener, Vincent Perreten, Andrea Endimiani

**Affiliations:** 1 Institute for Infectious Diseases, University of Bern, Bern, Switzerland; 2 Department of Infectious, Parasitic and Immune-Mediated Diseases, Istituto Superiore di Sanità, Rome, Italy; 3 Institute of Veterinary Bacteriology, Vetsuisse Faculty, University of Bern, Bern, Switzerland; 4 Graduate School for Cellular and Biomedical Sciences, University of Bern, Bern, Switzerland; Belgian Nuclear Research Centre SCK•CEN, BELGIUM

## Abstract

**Introduction:**

*bla*_OXA-48_, *bla*_NDM-1_ and *bla*_CTX-M-3_ are clinically relevant resistance genes, frequently associated with the broad-host range plasmids of the IncL/M group. The L and M plasmids belong to two compatible groups, which were incorrectly classified together by molecular methods. In order to understand their evolution, we fully sequenced four IncL/M plasmids, including the reference plasmids R471 and R69, the recently described *bla*_OXA-48_-carrying plasmid pKPN-El.Nr7 from a *Klebsiella pneumoniae* isolated in Bern (Switzerland), and the *bla*_SHV-5_ carrying plasmid p202c from a *Salmonella enterica* from Tirana (Albania).

**Methods:**

Sequencing was performed using 454 Junior Genome Sequencer (Roche). Annotation was performed using Sequin and Artemis software. Plasmid sequences were compared with 13 fully sequenced plasmids belonging to the IncL/M group available in GenBank.

**Results:**

Comparative analysis of plasmid genomes revealed two distinct genetic lineages, each containing one of the R471 (IncL) and R69 (IncM) reference plasmids. Conjugation experiments demonstrated that plasmids representative of the IncL and IncM groups were compatible with each other. The IncL group is constituted by the *bla*_OXA-48_-carrying plasmids and R471. The IncM group contains two sub-types of plasmids named IncM1 and IncM2 that are each incompatible.

**Conclusion:**

This work re-defines the structure of the IncL and IncM families and ascribes a definitive designation to the fully sequenced IncL/M plasmids available in GenBank.

## Introduction

IncL/M is currently one of the six major resistance plasmid families identified in clinically relevant *Enterobacteriaceae*, together with IncF, IncA/C, IncI, IncHI, and IncN [1]. IncL/M-type plasmids have been identified in association with antibiotic resistance genes encoding extended-spectrum β-lactamases (ESBL) (*bla*_CTX-M-3_, *bla*_SHV-5_), class A, B and D carbapenemases (*bla*_KPC_, *bla*_IMP_, *bla*_NDM_, and *bla*_OXA-48_), AmpC β-lactamases (*bla*_FOX-7_), as well as with the *armA* 16S rRNA methylase gene, conferring resistance to all aminoglycosides [2–11]. In particular, a unique epidemic IncL/M-type plasmid (pOXA-48) is at the origin of the worldwide dissemination of *bla*_OXA-48_ [9].

In previous studies, the comparative analysis of the variable region of fully sequenced IncL/M plasmids identified mobile elements that contributed to the acquisition of resistance genes, but scarce attention was paid to the plasmid backbones. However, it was noticed that the epidemic pOXA-48 plasmid showed significant backbone homology with other IncL/M plasmids except for three genes (*traX*, *traY* and *excA*) [7].

The target (ExcA) and exclusion (TraY) proteins are part of the so called “*entry exclusion system*” of conjugative plasmids [12]. During bacterial conjugation, plasmid DNA is transferred into a recipient cell via cell-to-cell contact. Transfer may be inhibited when donor and recipient cells harbor closely related conjugative plasmids. The interaction of ExcA in the recipient cell with its cognate TraY in the donor cell is one of the mechanisms that impairs the conjugation and inhibits redundant DNA transfer. Therefore, two cells carrying plasmids with different ExcA-TraY proteins should be able to successfully conjugate [13]. Furthermore, TraX is the presumptive relaxase of the plasmid and its DNA sequences have been used as molecular markers for plasmid classification [14]. Therefore, plasmids showing different *traX* genes should be classified in different phylogenetic groups.

Based on these observations, we hypothesized that there are two different plasmid types in the IncL/M family. This was also supposed in the 70s’, when two different groups (i.e., IncL and IncM) were initially defined by incompatibility tests performed by conjugation [15]. Later, in the early 80s’, IncL and IncM plasmids were combined into the IncL/M group, due to their high-level of DNA homology [16,17].

Because of the clinical and epidemiological relevance of these plasmid families, we fully sequenced the reference plasmids R471 and R69 formerly assigned by incompatibility assays to the IncL and IncM groups, respectively. Two recently isolated IncL/M plasmids carrying *bla*_SHV-5_ or *bla*_OXA-48_ were also fully sequenced for comparative analysis.

Based on the plasmid genome and conjugative properties of the plasmids, the present work re-defines the structure of the IncL and IncM families and assigns the fully sequenced IncL/M plasmids available in GenBank to their corresponding groups.

## Materials and Methods

### Bacterial strains

Plasmids R69 and R471 were from the collection of reference plasmids used for incompatibility testing available at the Istituto Superiore di Sanità, Rome, Italy [18]. Plasmid R69 was isolated in 1970 from *Salmonella paratyphi* B in France and assigned to the compatibility group com7, also named IncM [19]. Plasmid R471 was isolated in 1977 from *Serratia marcescens* in the USA and initially assigned to IncL, then re-classified as IncM [15,16]. Plasmid p202c was identified in *S*. *enterica* Typhimurium in 1985 in Albania and assigned to IncL/M group [3] by Southern blot hybridization [17] and PCR-Based Replicon Typing (PBRT; [18]). Plasmid pKPN-El-Nr.7 was isolated from *Klebsiella pneumoniae* AE-2247421 in 2013 in Switzerland [20] and assigned to IncL/M using the *repA* PCR devised on the pOXA-48 plasmid [9]. Plasmid pNDM-OM was identified in *K*. *pneumoniae* in 2010 in the Sultanate of Oman and fully sequenced [7] (Table 1).

**Table 1:** Fully sequenced plasmids belonging to the IncL and IncM families analyzed in this study.

Plasmid name	Plasmid size (base pairs)	GenBank Acc. No.	Resistance gene(s) on plasmid	Inc group ^a^	Plasmid core genome (nucleotide positions)	Plasmid core genome (length in base pairs)
pKPN-El-Nr.7	63,581	This study: KM406491	*bla*_OXA-48_	IncL	971–33389;58247–62497	36,668
pE71T	63,578	KC335143	*bla*_OXA-48_	IncL	26221–59607; 25453–20887	37,952
pKPoxa-48N1	62,592	NC_021488	*bla*_OXA-48_	IncL	1–26200;51044–62592	37,748
pKPoxa-48N2	167,203	NC_021502	*bla*_OXA-48_	IncL	1–26200; 155651–167230	37,748
pOXA48	61,881	NC_019154	*bla*_OXA-48_	IncL	19660–57449	37,789
R830b	81,793	NC_019344	*merA*	IncL	32333–68829	36,496
R471	86,748	This study: KM406489	*bla*_TEM-1_, *merA*	IncL	61219–86748;1–10968	36,497
pCTX-M3	89,168	NC_004464	*bla*_CTX-M-3_, *armA*, *dfrA12*, *aacC2*, *aadA2*, *sul1*, *mph*(E), *bla*_TEM-1_	IncM2	18187–55934	37,747
pCTX-M360	68,018	NC_011641	*bla*_CTX-M-3_, *bla*_TEM-1_	IncM2	1–26214; 56602–68018	37,630
pEl1573	87,731	NC_019368	*bla*_IMP-4_, *bla*_TEM-1_, *aacA4*, *catB3*, *mph*(A), *aacC2*, *qacG*, *sul1*, *qnrB2*	IncM2	15129–52921	37,792
pNDM-HK	83,803	NC_019063	*bla*_NDM-1_, *bla*_TEM-1_, *aacC2*, *armA*, *Δbla*_DHA-1_, *mph*(E), *sul1*	IncM2	52321–88803; 1–1594	38,076
pNDM-OM	87,185	NC_019889	*bla*_NDM-1_, *bla*_TEM-1_, *aacC2*, *armA*, *Δbla*_DHA-1_, *mph*(E), *sul1*	IncM2	975–39051	38,076
p202c	79,502	This study: KM406490	*bla*_SHV-5_, *tetA*(A), *aacC1*, *aacA4*, *aadA1*, *merA*	IncM1	4430–40962	36,532
pEL60	60,145	AY422214	No resistance genes	IncM1	14746–53148	38,402
R69	78,899	This study: KM406488	*bla*_TEM-1_, *tetA*(B), *aphA-1*, *merA*	IncM1	11238–47808	36,570
pNE1280	66,531	NC_019346	*bla*_KPC-4_	IncM1	1-2418655671-66531	35,046
pFOX-7a	90,439	HG934082	*bla*_FOX-7_, *bla*_TEM-1_, *aacA4*, *sul1*, *merA*	IncM1	1373–2983; 7932–44137	37,817

^a^ According to the new PBRT strategy proposed in this work.

All plasmids sequenced in this study were previously transferred into *E*. *coli* K12 strains by conjugation or transformation [3, 7, 20]. Plasmids R69 and R471 confer resistance to ampicillin/tetracycline/kanamycin and ampicillin, respectively. Plasmid p202c confers resistance to third-generation cephalosporins by the presence of the *bla*_SHV-5_ ESBL gene and carries further resistance determinants, including the In-t3 class 1 integron, containing aminoglycoside resistance gene cassettes [3, 21]. Plasmids pKPN-El-Nr.7 [20] and pNDM-OM [7] confer carbapenem resistance due to the expression of the *bla*_OXA-48_ and *bla*_NDM-1_ genes, respectively.

### Incompatibility assays

Incompatibility assays were performed crossing R69 x pKPN-El.Nr.7 and R69 x pNDM-OM *E*. *coli* transformants on solid Luria-Bertani (LB) agar plates without antibiotics. R69 x pKPN-El-Nr.7 transconjugants were selected on LB agar containing 50 μg/ml of kanamycin and 0.5 μg/ml of imipenem. Selected transconjugants were analyzed by plasmid DNA purification (see below).

Fourteen transconjugants were also tested for stability, by sub-cultivation in LB liquid medium without antibiotics for three days. An aliquot of each culture was plated daily on LB agar plates containing either no antibiotics, or containing 0.5 μg/ml of imipenem, or 50 μg/ml of kanamycin, or both 0.5 μg/ml imipenem and 50 μg/ml kanamycin. Fifty colonies from LB agar plates containing no antibiotics were screened for the presence of the IncL and IncM plasmids by PBRT (see below).

The R69 x pNDM-OM conjugation was first incubated 45 min at 37°C in 2 ml LB containing 1 μg/ml of tetracycline for the induction of the tetracycline resistant phenotype [22]. Appropriate dilutions of the induced cultures were then selected on solid LB agar plates containing 20 μg/ml of tetracycline and 0.5 μg/ml of imipenem.

### Restriction analysis of plasmid DNAs

R69, pKPN-El-Nr.7 and transconjugant plasmid DNAs were purified by the Genopure Plasmid Midi Kit (Roche Diagnostic, GmbH, Mannheim, Denmark) following the manufacturer’s protocol. Plasmid DNA was analyzed as undigested (data not shown) or restricted with BamHI, EcoRI and EcoRV (Bioconcept, Allschwil, Switzerland). Restriction patterns were analyzed on 0.8% agarose gel electrophoresis.

### Plasmid DNA sequencing

Complete DNA sequences of R69, R471, p202c and pKPN-El-Nr.7 were obtained following the 454-Junior Genome Sequencer procedure (Roche Diagnostic) on plasmid libraries. Plasmid DNA was purified from the respective *E*. *coli* transformants by the Genopure Plasmid Midi Kit (Roche Diagnostic).

### De novo assembly of DNA reads and gap-closure

Contigs with at least a 50-fold coverage were obtained using the GS-FLX gsAssembler software. Contigs were firstly assembled *in silico* by the 454 ReadStatus output file, generated by the gsAssembler software (Roche Diagnostics), identifying reads overlapping adjacent contigs. Contigs assembly and predicted gaps were then confirmed and filled by PCR-based gap closure by Sanger DNA sequencing of the amplicons.

### Annotation

Protein prediction was performed with Artemis Version 8 (Sanger Institute). Pairwise alignment was performed by a BLASTN and BLASTP homology search (http://blast.ncbi.nlm.nih.gov/Blast.cgi).

### Phylogenetic analysis of IncL/M plasmids

Homology and phylogenetic trees were obtained by aligning the IncL/M DNA sequences listed in Table 1. For the comparative analysis of the plasmid backbone, a core genome sequence was obtained as follows: for each plasmid, DNA sequences shared by all the IncL/M plasmids were identified by BLASTN and jointed to form a co-linear sequence of approximately 38 Kb, whose boundaries are indicated in Table 1. The alignment of core genomes was generated using the DNAMAN phylogenetic analysis software (Lynnon BioSoft, Vaudreuil, Quebec, Canada), for Quick Alignment. Unrooted phylogenetic trees were generated by the Maximum Likelihood method.

### PBRT update for the IncL and IncM plasmid families

IncL PBRT was performed using the L- FW (5'- CGG AAC CGA CAT GTG CCT ACT -3') and L/M- RV (5'- GAA CTC CGG CGA AAG ACC TTC-3') primer pair. The IncM PBRT was performed using M-FW (5'- GGA TGA AAA CTA TCA GCA TCT GAA G -3') and L/M- RV (5'- GAA CTC CGG CGA AAG ACC TTC-3') primers. The expected amplicons were 854 bp for the IncL and 738 bp for the IncM plasmids, respectively. The IncM forward primer is that previously reported in the IncL/M PBRT [18].

The PCR conditions were as follows: 1 cycle of denaturation at 94°C for 10 minutes, followed by 30 cycles of denaturation at 95°C for 1 minute, annealing at 60°C for 30 seconds and elongation at 72°C for 1 minute, followed by 1 cycle at 72°C for 5 minutes.

The IncL/M-RV primer was used for sequencing the PBRT amplicon, determining the IncM1 and IncM2 *inc*RNA type.

### Nucleotide sequence accession numbers

Complete nucleotide sequences of R69, R471, p202c and pKPN-El-Nr.7 have been deposited into the EMBL database under EMBL accession numbers KM406488, KM406489, KM406490, KM406491, respectively.

## Results and Discussion

### Plasmid incompatibility assay

**Fig 1:**
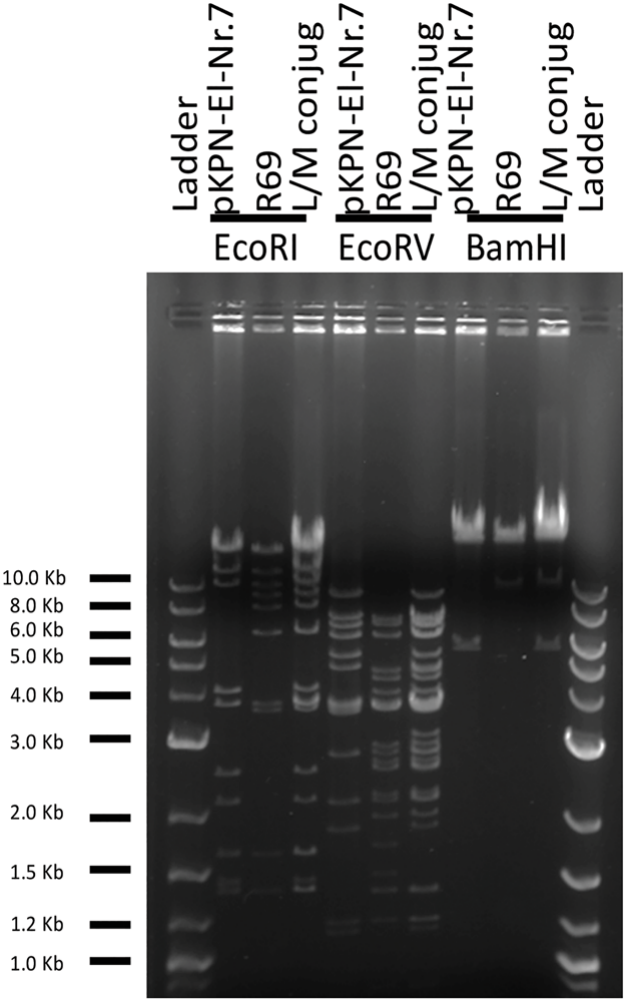
*Eco*RI, *Eco*RV and *Bam*HI restriction profiles of purified IncL/M plasmid DNAs, analyzed on 0.8% agarose gel. DNA on the gel: DNA ladder XV (Roche Diagnostic, GmbH, Mannheim, Germany); *E*. *coli* pKPN-El-Nr.7 plasmid; *E*. *coli* R69 (IncM reference plasmid); plasmid purified from the R69 x pKPN-El-Nr.7 transconjugant.

Incompatibility assays were performed using the *E*. *coli* transformants obtained from the IncM reference plasmid R69 and the *bla*_OXA-48_-positive pKPN-El-Nr.7 plasmid [20]. Transconjugants were obtained at very high frequency (2 x 10^-3^ transconjugants per donor cell). Plasmid DNAs of R69, pKPN-El-Nr.7 and one of the transconjugants were purified and restricted with BamHI, EcoRI and EcoRV. As expected, the transconjugant produced restriction patterns compatible with the co-presence of R69 and pKPN-El-Nr.7 in the same cell (Fig 1).

Stability experiments performed on transconjugants showed that >80% of the bacterial cells still showed resistance to both imipenem (marker of the pKPN-El-Nr.7 plasmid) and kanamycin (marker of the R69 plasmid) after three days of sub-culture without antibiotics. Therefore, transconjugants were stable even when cultivated in absence of antibiotic selective pressure. More importantly, these experiments demonstrated that the *bla*_OXA-48_-positive pKPN-El-Nr.7 plasmid was compatible with the IncM reference plasmid, and therefore it formally belongs to another group than IncM.

### Complete nucleotide sequence of IncL/M plasmids: the variable regions conferring antimicrobial resistance

**Fig 2:**
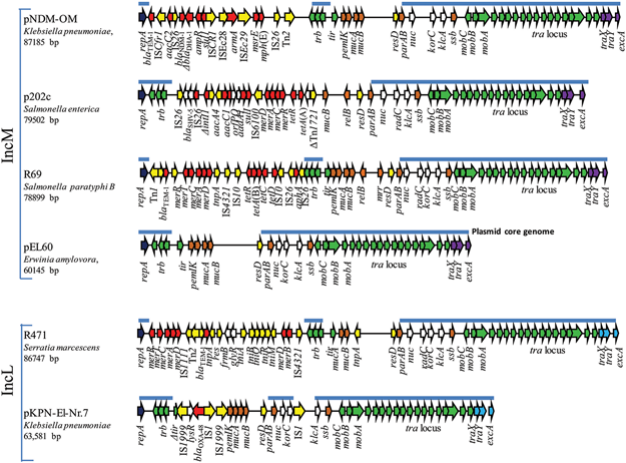
Major structural features of IncM and IncL plasmids. p202c, R69, R471 and pKPN-El-Nr.7 plasmids sequenced in this study are compared with pNDM-OM from *K*. *pneumoniae* 601 [7] and pEL60 [26]. In the in scale schematic representation, the open reading frames identified in the sequence are represented with arrows of different colors: the *tra* and *trb* transfer loci are in green; the *traX*, *traY*, *excA* entry exclusion genes of the IncM type are in purple, whereas those of the IncL type are in pale blue; resistance genes are in red; transposon-related genes [*tnpA*, *tnpR*, *tnpM*], insertion sequences, integrase and resolvase genes are in yellow; the replicase gene is in blue; partitioning genes, toxin-antitoxin and other stabilization systems are in brown; additional genes of unknown function are in white. The line above each plasmid represents the core genome used in comparative analysis.

The complete sequences of the four IncL/M plasmids showed that R69, R471, p202c, and pKPN-E1.Nr.7 were 78,899 bp, 86,747 bp, 79,505 bp and 63,581 bp length, respectively (Fig 2). In R471 and R69, the ampicillin resistance was due to the *bla*_TEM-1_ gene associated with the Tn*2* transposon in R471 and Tn*1* in R69, both flanked by the Tn*1696*-like *mer* operon conferring resistance to mercuric ions. R471 contains a complete Tn*402*-like transposon, including the *tni*, *tniB*, *tniC*, *tniR* and *tniM* genes. The tetracycline resistance on R69 was due to the presence of a complete Tn*10* composite transposon containing the *tetR*, *tetA*(B), *tetC*, *tetD* genes flanked by IS*10*. The kanamycin resistance determinant consisted of the Tn*6023* composite transposon, containing the IS*26*-*aphA*—IS*26* module (Fig 2).

The variable region of plasmid p202c contained the *bla*_SHV-5_ gene and the In-t3 class 1 integron carrying the *aacA4*, *aacC1*, orfP, orfQ, *aadA1* gene cassettes, whose *intI1* gene was truncated by the IS*26* insertion [3, 23]. On this plasmid, the Tn*1696 merA* operon and the ΔTn*1721* transposon carrying the tetracycline resistance gene *tetA*(A) were also identified (Fig 2).

pKPN-El-Nr.7 showed 100% nucleotide identity with the scaffold of other previously sequenced plasmids carrying the *bla*_OXA-48_ gene. The carbapenemase gene was mobilized by the Tn*1999*.*2* transposon, including the IS1R element integrated in the IS*1999* located upstream of the *bla*_OXA-48_ gene [24]. No resistance genes other than *bla*_OXA-48_ were identified on this plasmid, as previously described for the pOXA-48 plasmid and its relatives (Table 1).

### Core genome of IncL/M plasmids: designation of separate IncL and IncM plasmid families

BLASTN comparative analysis was performed on the 4 plasmids sequenced in this study and 13 fully sequenced plasmids available in GenBank (Table 1). The most conserved part of the IncL/M plasmid backbone encoded the transfer loci (*tra*), the replicon (the *repA* replicase gene was highly conserved among L and M plasmids, showing >99% nucleotide identity), the partitioning module (*parA*, *parB*), and a large number of hypothetical proteins [4, 25, 26]. The exclusion system (*excA*, *traY*) and relaxase (*traX*) genes were present in all plasmids, but showed diverging sequences. Toxin-antitoxin systems, *mucA-mucB* genes and resistance determinants were not present in all plasmids (Fig 2).

DNA sequences corresponding to regions shared by all plasmids were jointed to produce a co-linear sequence of approximately 38 Kb representing the core genome of the IncL/M plasmids (Table 1).

**Fig 3:**
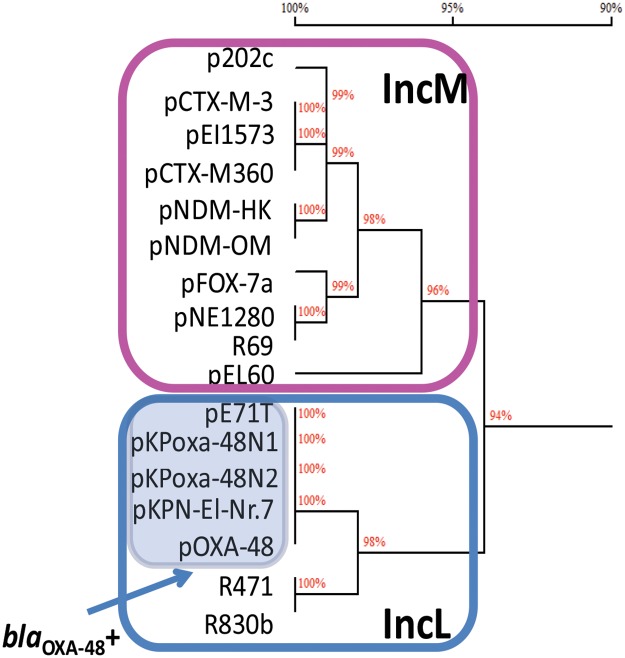
Homology trees of the IncL and IncM core genomes. Core genomes contain the entire scaffold, excluding antimicrobial resistance determinants and genes that were not present in all the plasmids under study. The percentage of nucleotide identity among the compared plasmid core genomes is shown on each branch of the trees. Panels highlight the IncM and IncL branch separation on the tree. The plasmids positive for the *bla*_OXA-48_ gene are highlighted by a pale blue panel.

**Fig 4:**
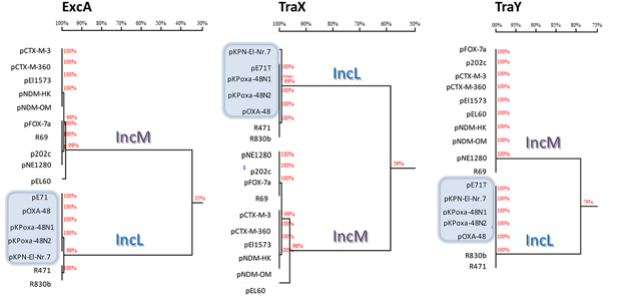
Homology trees of the ExcA, TraX, TraY protein sequences. The deduced protein sequences of the proteins from each respective plasmid were downloaded from the GenBank or deduced from the DNA sequences of plasmids performed in this study. The percentage of amino acid identity among the compared protein sequences is shown on each branch of the trees. Plasmids positive for the *bla*_OXA-48_ gene are highlighted by pale blue panels.

The comparative analysis demonstrated high level of DNA homology (overall nucleotide identity of approximately 94%). However, the homology tree clearly revealed the presence of two branches, suggesting the presence of two different plasmid backbones (IncL and IncM; Fig 3). This division was evident comparing ExcA, TraY, and TraX proteins (35%, 59%, and 75% amino acid identity, respectively) (Fig 4).

**Fig 5:**
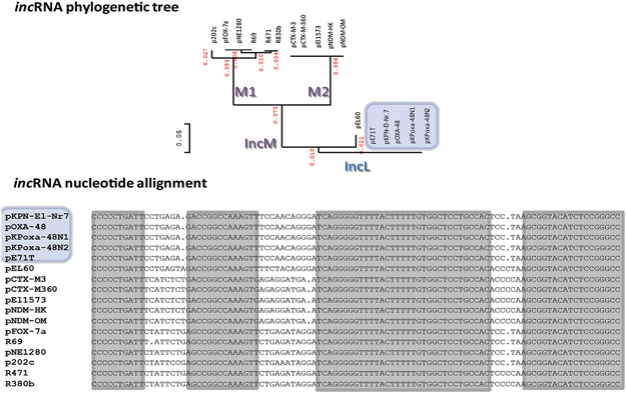
Phylogenetic tree and nucleotide sequence alignment of the major stem-and-loop *inc*RNA of IncL and IncM plasmid. The *inc*RNA sequences were downloaded from the GenBank or identified in plasmids sequenced in this study. The unrooted phylogenetic tree inferred the evolutionary relationships among the various *inc*RNAs based upon similarities and differences in their nucleotide sequences. The part of the phylogeny tree corresponding to plasmids positive for the *bla*_OXA-48_ gene is highlighted by pale blue panels. In the panel showing the nucleotide alignment, the residues showing 100% identity have been shaded. The white parts of the alignment show the mismatches identified among the IncL and IncM *inc*RNA sequences.

The *inc*RNA, encoding the antisense RNA regulating the *repA* gene expression, has been described as the molecular base of the incompatibility behavior of plasmids belonging to the IncFII-complex, also including IncFII, IncI, IncK, IncB/O and IncL/M [27]. The *inc*RNA of the IncL/M plasmids demonstrated high heterogeneity. In particular, three major branches were observed by phylogenetic analysis: one corresponding to IncL and two belonging to IncM (Fig 5).

### IncL plasmids

This study assigns pOXA-48 and its relatives to the IncL group. These plasmids show unique exclusion system and *inc*RNA sequences, which suggest broad compatibility with other plasmids, including the more closely related IncM. The genetic analysis of plasmid DNA sequences supported the experimental evidence obtained by conjugation, demonstrating that pOXA-48 is fully compatible with the IncM R69 plasmid.

The analysis suggests an evolutionary model in which all IncL/M-type plasmids have diverged from a common ancestor through the acquisition of different entry exclusion systems and relaxases and differentiation of the *inc*RNA determinant in the replicon. The evolution process also involved the integration of the *bla*_OXA-48_ mobile element conferring carbapenem resistance. It has been demonstrated that the integration of Tn*1999* in the *tir* gene increases the conjugation performance of pOXA-48 [28]. These peculiar conjugative features may explain the great success and the current worldwide spread of the pOXA-48-like plasmids.

### IncM plasmids

Plasmids pNDM-OM, pCTX-M-3, pCTXM-360, pNDM-HK, pEl1573, pNE1280, p202c and pEL60 were all highly homologous to R69 and are here designated as IncM members. IncM plasmids show the same backbone but substantially differ in resistance modules (Table 1, Fig 2). The unrooted phylogenetic tree obtained comparing the *inc*RNA sequences suggested that two types of IncM replicons here designated as M1 and M2 exist (Fig 5). To check the incompatibility behavior of IncM1 and IncM2 plasmids, conjugation assays were performed crossing R69 (IncM1) with pNDM-OM (IncM2). Transconjugants were not obtained in our conditions (limit of detection lower that 1 x 10^-6^), on LB agar plates containing tetracycline (marker for R69) and imipenem (marker for pNDM-OM). This result was expected because of the presence on both plasmids of the same entry exclusion ExcA and TraY proteins (99% amino acid identity between IncM1 and IncM2). The entry exclusion system is efficiently preventing the conjugation among IncM1 and IncM2 plasmids. However, the divergence of the *inc*RNA is an interesting marker for plasmid typing to identify related lineages within the IncM group.

### PCR-Based Replicon Typing (PBRT) of the IncL and IncM groups

Since the epidemiological relevance of the members of these plasmid families and the difficulty to distinguish correctly IncM and IncL plasmids by the current methods, the PBRT method has been updated. Using the former PBRT version, IncM plasmids were typable but not those IncL and pOXA-48-like. This was due to the forward primer which was designed considering only the *excA* gene of the IncM plasmid R69 but not that of IncL plasmids (e.g., R471) [18]. Recently, it has been proposed to use the *repA*, *parA* and *traU* genes as markers for the detection of all IncL/M plasmids [9], but these primers are not able to differentiate between the different IncL/M groups. Therefore, two new forward primers have been designed on the *excA* gene of the IncL [L- FW] and IncM plasmid types [M-FW], respectively. These primers are used with a unique reverse primer [L/M- RV], targeting the highly conserved *repA* gene of both IncL and IncM plasmids.

Based on the new primer strategy, the new PBRT scheme demonstrated 100% specificity and sensitivity in the detection and discrimination of the IncL and IncM replicons. The specificity of this test was verified on 20 plasmids belonging to IncA/C, IncFIIK, IncF, IncHI1, IncHI2, IncI1, IncI2, IncN, IncT, IncX2, IncX1, IncU groups and on 6 belonging to the former IncL/M group (Table 2) [29–44].

**Table 2:** PCR-Based Replicon Typing (PBRT) for IncL and IncM plasmids: list of plasmids used for the specificity assay.

Plasmid name	Reference	Incompatibility group previously assigned by conjugation and/or PBRT	PBRT IncL	PBRT IncM
R69	[19]	IncL/M	negative	positive
p202c	[3]	IncL/M	negative	positive
pNDM-OM	[7]	IncL/M	negative	positive
pKPN-El-Nr.7	[20]	IncL/M	positive	negative
R471	[16]	IncL/M	positive	negative
Sk-1	[29]	IncL/M	positive	negative
p2039	[30]	IncA/C	negative	negative
R387	[31]	IncK	negative	negative
366-D2	[32]	IncFIme	negative	negative
R100	[32]	IncFII	negative	negative
LS6	[33]	IncFIIK1	negative	negative
pKPN3-IT	[34]	IncFIIK2	negative	negative
R6k	[35]	IncX2	negative	negative
pOLA52	[36]	IncX1	negative	negative
pRA3	[37]	IncU	negative	negative
172.23(T)	[37]	IncR	negative	negative
pOXA-181	[38]	IncT	negative	negative
R46	[39]	IncN	negative	negative
p1358	[37]	IncI1-α	negative	negative
R483	[18]	IncI1-α	negative	negative
R621a	[18]	IncI1-γ	negative	negative
pNDM-MAR	[40]	IncH-like	negative	negative
R478	[41]	IncHI2	negative	negative
759-D	[42]	IncHI2-FII-FIB	negative	negative
R27	[43]	IncHI1	negative	negative
pEQ2	[44]	IncHI1-X1	negative	negative

The identification of the IncM1 and IncM2 sub-types can be performed by sequencing the IncM PBRT amplicon obtained as described above, using the L/M-RV primer. In fact, this amplicon includes the *inc*RNA sequence discriminating the M1 and M2 replicons.

## Conclusions

The IncM pCTX-M-3 plasmid exhibits very broad host-range, including α-, β- and γ-proteobacteria, able to replicate in *Agrobacterium tumefaciens*, *Ralstonia eutropha*, and *Pseudomonas aeruginosa* [25]. It was also demonstrated that pOXA-48 replicates in *Shewanella oneidensis*, a species close to the progenitor of the *bla*_OXA-48_ gene [45]. These findings suggest that both IncL and IncM plasmids may constitute an important interspecies vehicle for the dissemination of life-threatening resistance genes.

Conjugation and whole plasmid sequencing demonstrated that IncL and IncM plasmids, each carrying specific ExcA-TraY entry exclusion proteins, relaxases and incompatibility determinants, constitute the currently titled IncL/M family. The IncL group includes the reference plasmid R471, as well as the pOXA-48-like and R830b plasmids. The heterogeneity of the IncM group consisted in variable resistance modules and in the presence of divergent *inc*RNAs. However, conjugation between isolates carrying members of the IncM group was impaired by the inhibitory interaction of the exclusion system, which is highly conserved among all the IncM plasmids. IncL/M represents a nice example of plasmid evolution and differentiation, explaining the different levels of negative controls regulating the incompatibility behavior of highly related plasmids. The divergence of the *inc*RNA sequence is likely the first step for the differentiation of a new Inc group, but the presence of almost identical exclusion systems may still interfere with conjugation. The acquisition of a divergent exclusion system, such as that identified on the IncL plasmids, is the further step required to obtain full compatibility among related plasmids.
